# The genome sequence of the scale worm, *Lepidonotus clava *(Montagu, 1808)

**DOI:** 10.12688/wellcomeopenres.18660.1

**Published:** 2022-12-21

**Authors:** Teresa Darbyshire, John Bishop, Nova Mieszkowska, Patrick Adkins, Anna Holmes, Marine Biological Association Genome Acquisition Lab, Darwin Tree of Life Barcoding collective, Wellcome Sanger Institute Tree of Life programme, Wellcome Sanger Institute Scientific Operations: DNA Pipelines collective, Tree of Life Core Informatics collective, Darwin Tree of Life Consortium

**Affiliations:** 1 Amgueddfa Cymru, Cardiff, UK; 2 Marine Biological Association, Plymouth, Devon, UK

**Keywords:** Lepidonotus clava, scale worm, genome sequence, chromosomal, Polychaeta

## Abstract

We present a genome assembly from an individual *Lepidonotus clava* (scale worm; Annelida; Polychaeta; Phyllodocida; Polynoidae). The genome sequence is 1,044 megabases in span. Most of the assembly is scaffolded into 18 chromosomal pseudomolecules. The mitochondrial genome has also been assembled and is 15.6 kilobases in length.

## Species taxonomy

Eukaryota; Metazoa; Spiralia; Lophotrochozoa; Annelida; Polychaeta; Errantia; Phyllodocida; Polynoidae; *Lepidonotus*; *Lepidonotus clava* (Montagu, 1808) (NCBI txid:1210411).

## Background

*Lepidonotus clava* (Montagu, 1808) is a large-bodied (up to 45 mm), robust scale worm in the sub-family Lepidonotinae of Family Polynoidae. It was first described from the South Devon coast in England and is distributed around the UK and Ireland. Further afield, it is widely recorded along other European North Sea coasts, the Mediterranean, Red Sea and the west coast of Africa as well as the North Pacific ( Barnich & Fiege, 2003; Chambers & Muir, 1997; Parapar *et al.*, 2015). It is typically found on hard substrates ( Chambers & Muir, 1997), but is also known from a variety of other habitats including muddy and sandy substrates, algal holdfasts, seagrass beds and mussel beds ( Parapar *et al.*, 2015) and is found from the littoral down to 160 m ( Fauvel, 1936). It is not considered to be under threat or invasive as a non-native species anywhere in the world.

*Lepidonotus* species all have lateral antennae inserted terminally, 12 pairs of elytra and a smooth body. The 12 pairs of leathery elytra on *L. clava* are attached firmly to the body and are not shed easily as in some other scale worms. *L. clava* can be distinguished from other UK and European *Lepidonotus* by the elytra (surface covered with conical macro- and microtubercles and margin with no fringing papillae) and the neurochaetae all having unidentate tips. *L. clava* are carnivores, utilising a muscular evertible pharynx armed with a pair of jaws ( Jumars *et al.*, 2015), and are known to feed on other polynoids ( Chambers & Muir, 1997). They are gonochoric and females with eggs have been reported from the west coast of Scotland in February and July ( Chambers & Muir, 1997).

The genome of *L. clava* was sequenced as part of the Darwin Tree of Life Project, a collaborative effort to sequence all named eukaryotic species in the Atlantic Archipelago of Britain and Ireland.

## Genome sequence report

**Figure 1.**
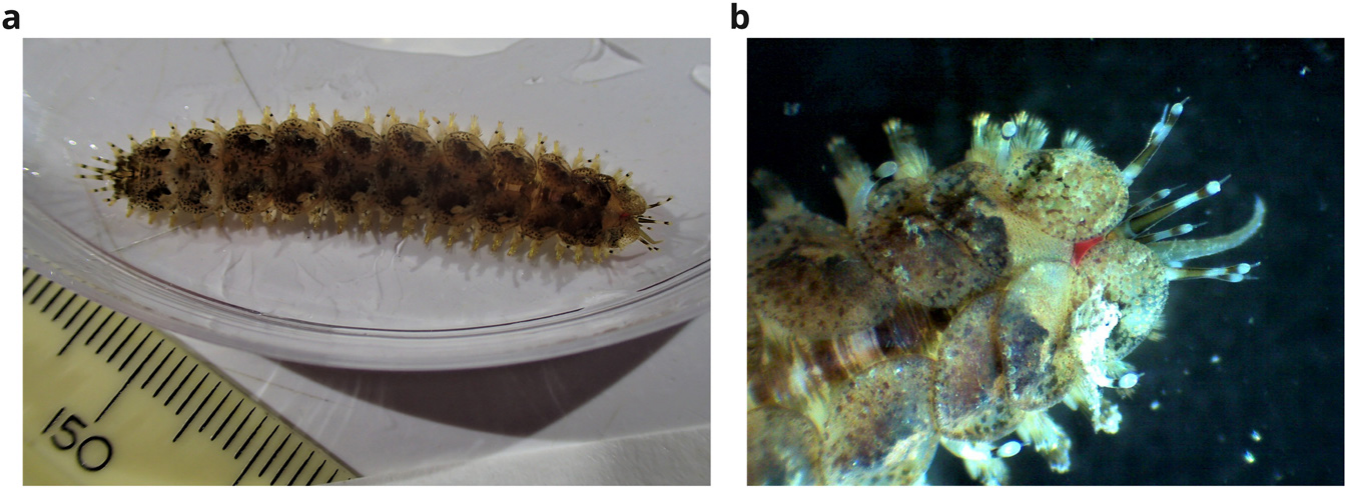
Photographs of the *Lepidonotus clava* specimen (wpLepClav1) taken immediately prior to dissection and preservation of tissue for whole genome sequencing, showing **a**) the dorsum **b**) the head (dorsal view).

The genome was sequenced from a single *L. clava* ( Figure 1) collected from Batten Bay (latitude 50.3554, longitude –4.1251). A total of 48-fold coverage in Pacific Biosciences single-molecule HiFi long reads and 32-fold coverage in 10X Genomics read clouds was generated. Primary assembly contigs were scaffolded with chromosome conformation Hi-C data. Manual assembly curation corrected 300 missing joins or misjoins and removed 41 haplotypic duplications, reducing the assembly length by 3.27% and the scaffold number by 33.88%, and increasing the scaffold N50 by 3.14%.

**Figure 2.**
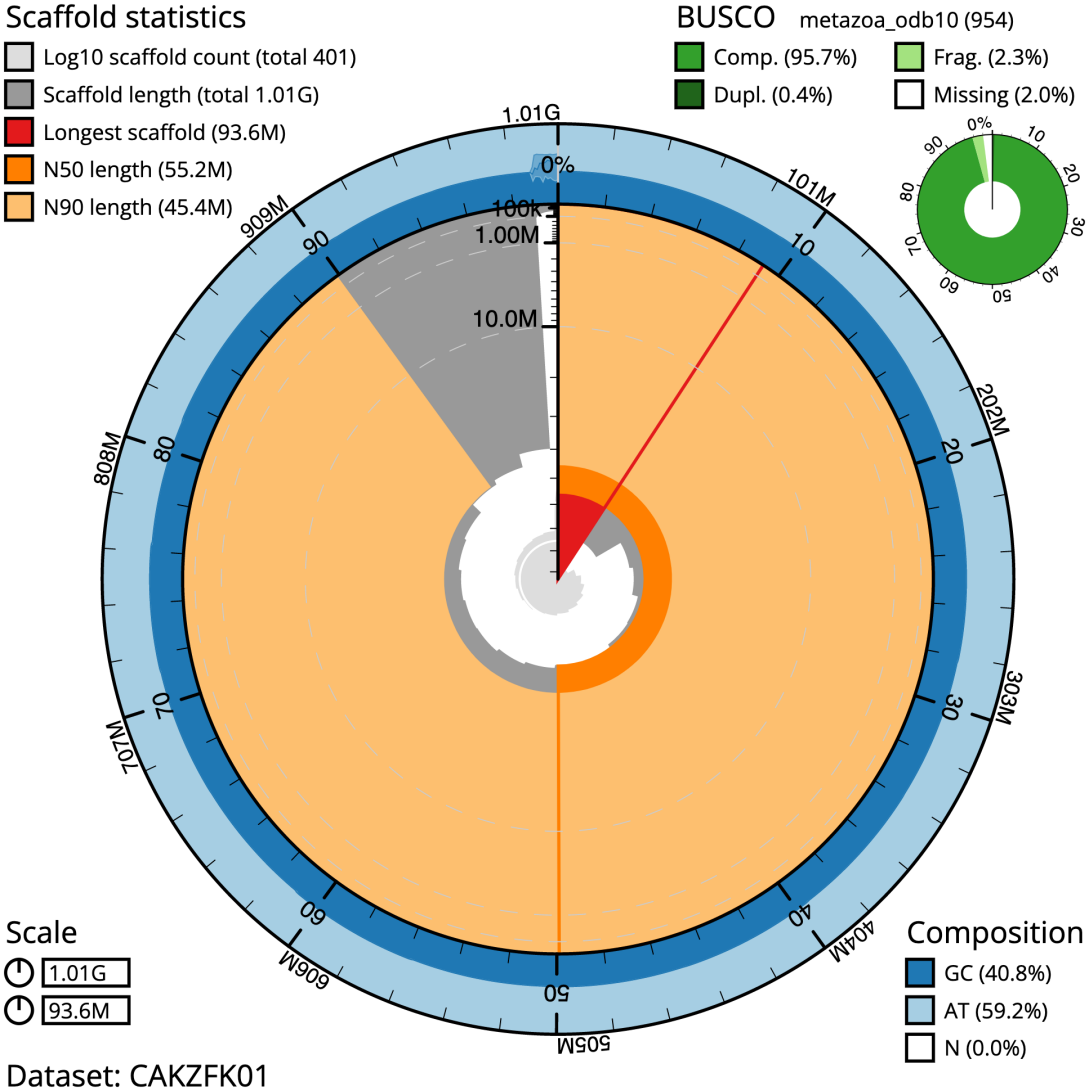
Genome assembly of *Lepidonotus clava,* wpLepClav1.1: metrics. The BlobToolKit Snailplot shows N50 metrics and BUSCO gene completeness. The main plot is divided into 1,000 size-ordered bins around the circumference with each bin representing 0.1% of the 1,010,252,647 bp assembly. The distribution of scaffold lengths is shown in dark grey with the plot radius scaled to the longest scaffold present in the assembly (93,563,122 bp, shown in red). Orange and pale-orange arcs show the N50 and N90 scaffold lengths (55,150,419 and 45,382,685 bp), respectively. The pale grey spiral shows the cumulative scaffold count on a log scale with white scale lines showing successive orders of magnitude. The blue and pale-blue area around the outside of the plot shows the distribution of GC, AT and N percentages in the same bins as the inner plot. A summary of complete, fragmented, duplicated and missing BUSCO genes in the metazoa_odb10 set is shown in the top right. An interactive version of this figure is available at https://blobtoolkit.genomehubs.org/view/wpLepClav1.1/dataset/CAKZFK01/snail.

The final assembly has a total length of 1,010 Mb in 400 sequence scaffolds with a scaffold N50 of 55 Mb ( Table 1). Most (98.94%) of the assembly sequence was assigned to 18 chromosomal-level scaffolds, named in order of size ( Figure 2– Figure 5; Table 2). Repetitive scaffolds have been inserted into chromosome 15 (~13.7 Mb), and the order and orientation of these scaffolds is unclear. The assembly has a BUSCO v5.3.2 ( Manni *et al.*, 2021) completeness of 95.7% (single 95.3%, duplicated 0.4%) using the metazoa_odb10 reference set. While not fully phased, the assembly deposited is of one haplotype. Contigs corresponding to the second haplotype have also been deposited.

**Table 1.** Genome data for *Lepidonotus clava*, wpLepClav1.

Project accession data	
Assembly identifier	wpLepClav1.1
Species	*Lepidonotus clava*
Specimen	wpLepClav1
NCBI taxonomy ID	1210411
BioProject	PRJEB50789
BioSample ID	SAMEA8724790
Isolate information	Muscle tissue from the mid-body
Assembly metrics **	
Base pair QV	51.3 (Benchmark: ≥50)
*k*-mer completeness	99.98% (Benchmark: ≥95%)
BUSCO **	C:95.7%[S:95.3%,D:0.4%],F:2.3%, M:2.0%,n:954 (Benchmark: C ≥ 95%)
Percentage of assembly mapped to chromosomes	98.94% (Benchmark: ≥95%
Sex chromosomes	not identified (Benchmark: localised homologous pairs)
Organelles	Mitochondrial genome 15.6 kb (Benchmark: complete single alleles)
Raw data accessions	
PacificBiosciences SEQUEL II	ERR8575393–ERR8575395
10X Genomics Illumina	ERR8571687–ERR8571690
Hi-C Illumina	ERR8571691
PolyA RNA-Seq Illumina	ERR10123672
Genome assembly	
Assembly accession	GCA_936440205.1
Accession of alternate haplotype	GCA_936448955.1
Span (Mb)	1,010
Number of contigs	798
Contig N50 length (Mb)	7
Number of scaffolds	400
Scaffold N50 length (Mb)	55
Longest scaffold (Mb)	94

* Assembly metric benchmarks are adapted from column VGP-2020 of “Table 1: Proposed standards and metrics for defining genome assembly quality” from ( Rhie *et al.*, 2021). ** BUSCO scores based on the metazoa_odb10 BUSCO set using v5.3.2. C = complete [S = single copy, D = duplicated], F = fragmented, M = missing, n = number of orthologues in comparison. A full set of BUSCO scores is available https://blobtoolkit.genomehubs.org/view/wpLepClav1.1/dataset/CAKZFK01/busco.

**Figure 3.**
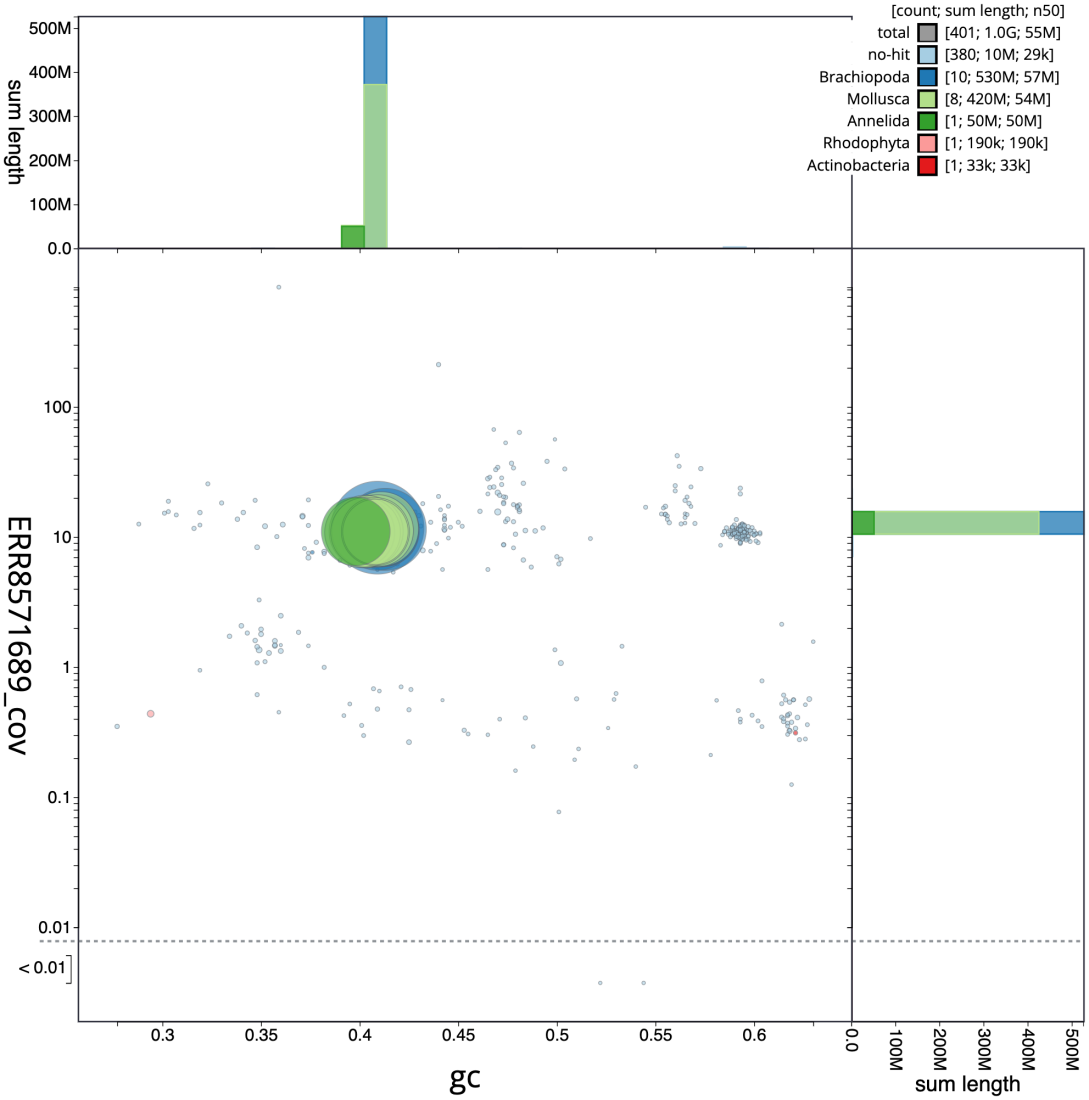
Genome assembly of *Lepidonotus clava,* wpLepClav1.1: GC coverage. BlobToolKit GC-coverage plot. Scaffolds are coloured by phylum. Circles are sized in proportion to scaffold length. Histograms show the distribution of scaffold length sum along each axis. An interactive version of this figure is available at https://blobtoolkit.genomehubs.org/view/wpLepClav1.1/dataset/CAKZFK01/blob.

**Figure 4.**
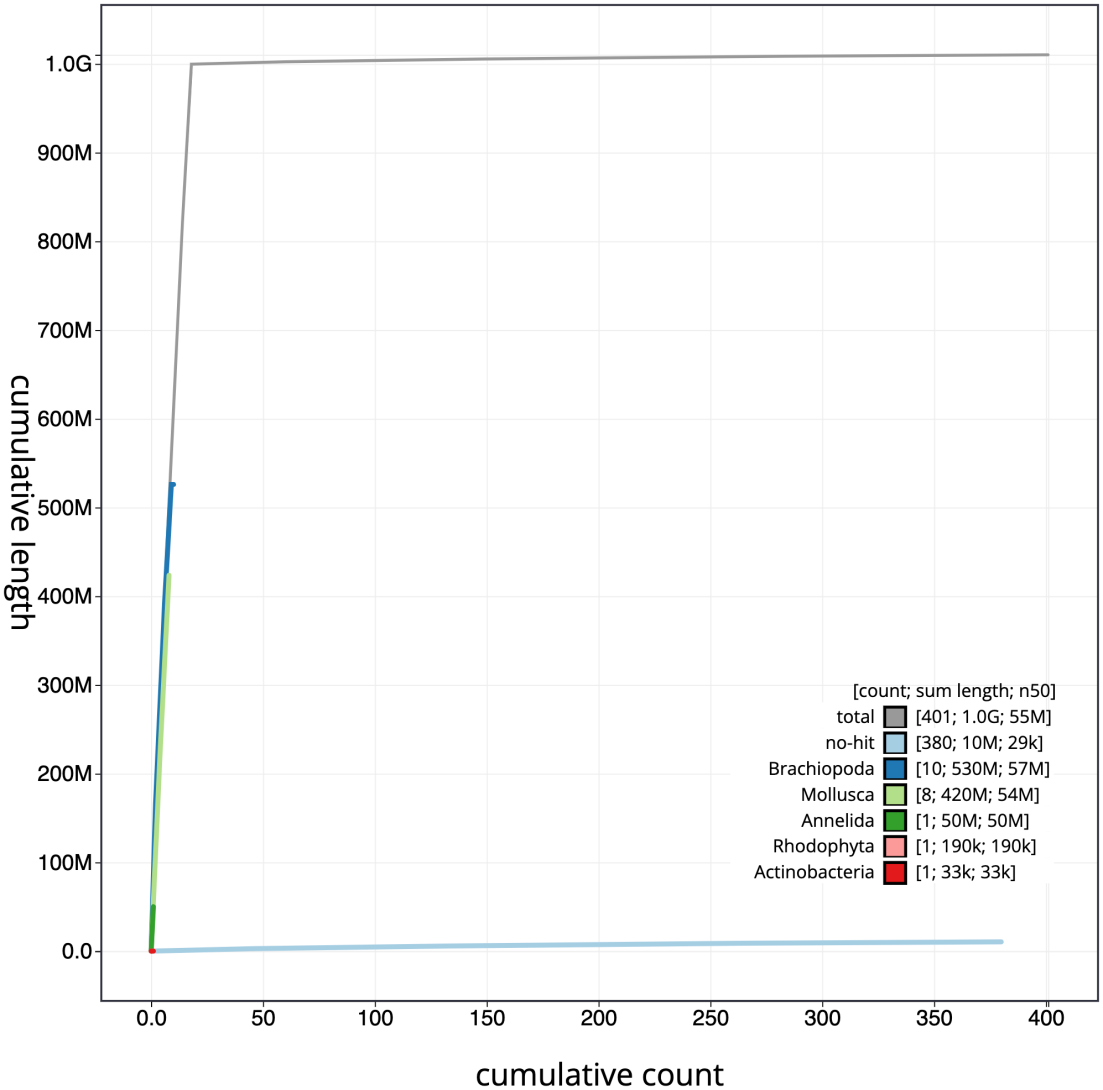
Genome assembly of *Lepidonotus clava,* wpLepClav1.1: cumulative sequence. BlobToolKit cumulative sequence plot. The grey line shows cumulative length for all scaffolds. Coloured lines show cumulative lengths of scaffolds assigned to each phylum using the buscogenes taxrule. An interactive version of this figure is available at https://blobtoolkit.genomehubs.org/view/wpLepClav1.1/dataset/CAKZFK01/cumulative.

**Figure 5.**
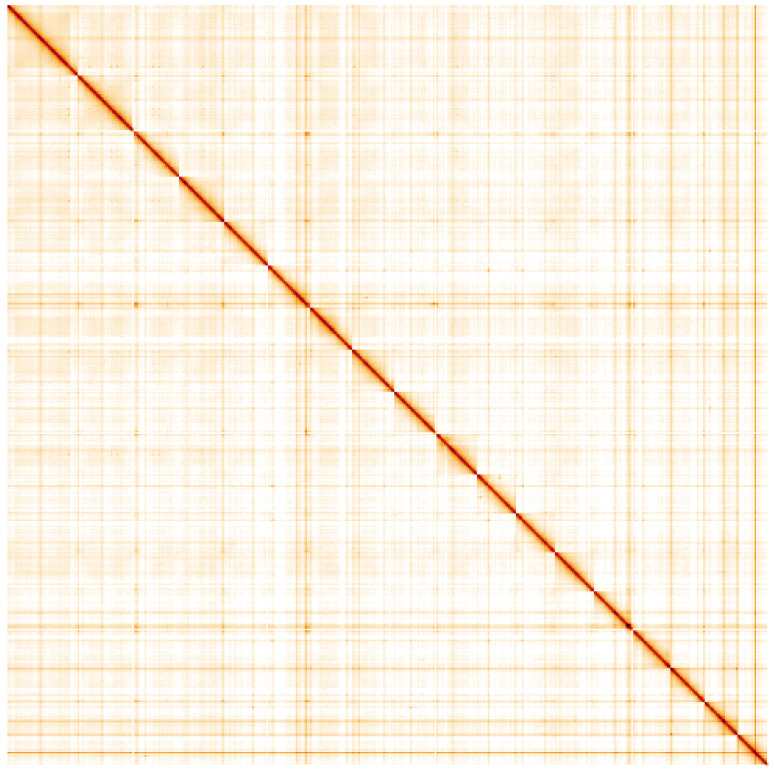
Genome assembly *Lepidonotus clava,* wpLepClav1.1: Hi-C contact map. Hi-C contact map of the wpLepClav1.1 assembly, visualised using HiGlass. Chromosomes are shown in order of size from left to right and top to bottom. An interactive version of this figure may be viewed at https://genome-note-higlass.tol.sanger.ac.uk/l/?d=eUaRZzx6TJ-22DPb6LlLvg.

**Table 2.** Chromosomal pseudomolecules in the genome assembly of *Lepidonotus clava*, wpLepClav1.

INSDC accession	Type	Size (Mb)	GC%
OW387133.1	1	93.56	40.9
OW387134.1	2	72.45	41.3
OW387135.1	3	60.62	40.9
OW387136.1	4	59.2	41.1
OW387137.1	5	56.84	40.3
OW387138.1	6	56.55	40.6
OW387139.1	7	55.32	41.2
OW387140.1	8	55.15	40.9
OW387141.1	9	54.02	40.4
OW387142.1	10	53.11	41.2
OW387143.1	11	51.98	40
OW387144.1	12	51.73	40.3
OW387145.1	13	51.09	41
OW387146.1	14	50.06	39.8
OW387147.1	15	49.31	40.8
OW387148.1	16	45.38	40.8
OW387149.1	17	43.84	40.3
OW387150.1	18	39.41	40.6
OW387151.1	MT	0.02	35.9

## Methods

### Sample acquisition and nucleic acid extraction

An individual adult *L. clava* (wpLepClav1) was collected from Batten Bay, Mount Batten, Devon, UK (latitude 50.3554, longitude –4.1251) by John Bishop, Nova Mieszkowska and Patrick Adkins, (all Marine Biological Association), and Teresa Derbyshire and Anna Holmes (both National Museum Wales). The specimen was identified by Teresa Derbyshire by means of macroscopic and microscopic morphology and DNA barcoding (COI). The sample was taken from a rock crevice by hand and preserved by freezing in liquid nitrogen.

DNA was extracted at the Tree of Life laboratory, Wellcome Sanger Institute. The wpLepClav1 sample was weighed and dissected on dry ice with tissue set aside for Hi-C sequencing. Muscle tissue was disrupted using a Nippi Powermasher fitted with a BioMasher pestle. Fragment size analysis of 0.01–0.5 ng of DNA was then performed using an Agilent FemtoPulse. High molecular weight (HMW) DNA was extracted using the Qiagen MagAttract HMW DNA extraction kit. Low molecular weight DNA was removed from a 20-ng aliquot of extracted DNA using 0.8X AMpure XP purification kit prior to 10X Chromium sequencing; a minimum of 50 ng DNA was submitted for 10X sequencing. HMW DNA was sheared into an average fragment size of 12–20 kb in a Megaruptor 3 system with speed setting 30. Sheared DNA was purified by solid-phase reversible immobilisation using AMPure PB beads with a 1.8X ratio of beads to sample to remove the shorter fragments and concentrate the DNA sample. The concentration of the sheared and purified DNA was assessed using a Nanodrop spectrophotometer and Qubit Fluorometer and Qubit dsDNA High Sensitivity Assay kit. Fragment size distribution was evaluated by running the sample on the FemtoPulse system. 

RNA was extracted from mid-body tissue of wpLepClav1 in the Tree of Life Laboratory at the WSI using TRIzol, according to the manufacturer’s instructions. RNA was eluted in 50 μl RNAse-free water and its concentration was assessed using a Nanodrop spectrophotometer and Qubit Fluorometer using the Qubit RNA Broad-Range (BR) Assay kit. Analysis of the integrity of the RNA was done using Agilent RNA 6000 Pico Kit and Eukaryotic Total RNA assay.

### Sequencing

Pacific Biosciences HiFi circular consensus and 10X Genomics read cloud DNA sequencing libraries were constructed according to the manufacturers’ instructions. Poly(A) RNA-Seq libraries were constructed using the NEB Ultra II RNA Library Prep kit. DNA and RNA sequencing was performed by the Scientific Operations core at the WSI on Pacific Biosciences SEQUEL II (HiFi), Illumina NovaSeq 6000 (DNA 10X and RNA-Seq) instruments. Hi-C data were also generated from mid-body tissue of wpLepClav1 using the Arima v2 kit and sequenced on the Illumina NovaSeq 6000 instrument.

### Genome assembly

Assembly was carried out with Hifiasm ( Cheng *et al.*, 2021) and haplotypic duplication was identified and removed with purge_dups ( Guan *et al.*, 2020). One round of polishing was performed by aligning 10X Genomics read data to the assembly with Long ranger ALIGN, calling variants with freebayes ( Garrison & Marth, 2012). The assembly was then scaffolded with Hi-C data ( Rao *et al.*, 2014) using YaHS ( Zhou *et al.*, 2022). The assembly was checked for contamination and corrected using the gEVAL system ( Chow *et al.*, 2016) as described previously ( Howe *et al.*, 2021). Manual curation ( Howe *et al.*, 2021) was performed using gEVAL, HiGlass ( Kerpedjiev *et al.*, 2018) and Pretext ( Harry, 2022). The mitochondrial genome was assembled using MitoHiFi ( Uliano-Silva *et al.*, 2021), which performed annotation using MitoFinder ( Allio *et al.*, 2020). The genome was analysed and BUSCO scores generated within the BlobToolKit environment (Challis *et al*.). Table 3 contains a list of all software tool versions used, where appropriate.

**Table 3.** Software tools used.

Software tool	Version	Source
BlobToolKit	3.4.0	Challis *et al.*, 2020
freebayes	1.3.1-17-gaa2ace8	Garrison & Marth, 2012
Hifiasm	0.15.3	Cheng *et al.*, 2021
HiGlass	1.11.6	Kerpedjiev *et al*., 2018
Long ranger ALIGN	2.2.2	https://support.10xgenomics.com/genome-exome/ software/pipelines/latest/advanced/other-pipelines
MitoHiFi	2.0	Uliano-Silva *et al.*, 2021
PretextView	0.2.x	Harry, 2022
purge_dups	1.2.3	Guan *et al.*, 2020
YaHS	1.0	Zhou *et al.*, 2022

### Ethics/compliance issues

The materials that have contributed to this genome note have been supplied by a Darwin Tree of Life Partner. The submission of materials by a Darwin Tree of Life Partner is subject to the Darwin Tree of Life Project Sampling Code of Practice. By agreeing with and signing up to the Sampling Code of Practice, the Darwin Tree of Life Partner agrees they will meet the legal and ethical requirements and standards set out within this document in respect of all samples acquired for, and supplied to, the Darwin Tree of Life Project. Each transfer of samples is further undertaken according to a Research Collaboration Agreement or Material Transfer Agreement entered into by the Darwin Tree of Life Partner, Genome Research Limited (operating as the Wellcome Sanger Institute), and in some circumstances other Darwin Tree of Life collaborators.

## Data Availability

European Nucleotide Archive: *Lepidonotus clava* (a scale worm). Accession number PRJEB50789; https://identifiers.org/ena.embl/PRJEB50789 ( Wellcome Sanger Institute, 2022) The genome sequence is released openly for reuse. The *Lepidonotus clava* genome sequencing initiative is part of the Darwin Tree of Life (DToL) project. All raw sequence data and the assembly have been deposited in INSDC databases. The genome will be annotated using available RNA-Seq data and presented through the Ensembl pipeline at the European Bioinformatics Institute. Raw data and assembly accession identifiers are reported in Table 1.

## Author information

Members of the Marine Biological Association Genome Acquisition Lab are listed here: https://doi.org/10.5281/zenodo.4783604.

Members of the Darwin Tree of Life Barcoding collective are listed here: https://doi.org/10.5281/zenodo.4893703.

Members of the Wellcome Sanger Institute Tree of Life programme are listed here: https://doi.org/10.5281/zenodo.4783585.

Members of Wellcome Sanger Institute Scientific Operations: DNA Pipelines collective are listed here: https://doi.org/10.5281/zenodo.4790455.

Members of the Tree of Life Core Informatics collective are listed here: https://doi.org/10.5281/zenodo.5013541.

Members of the Darwin Tree of Life Consortium are listed here: https://doi.org/10.5281/zenodo.4783558.
