# Supplementary material for: The genome sequence of the scale worm, Lepidonotus clava (Montagu, 1808)
Source: Wellcome Open Res. 2022 Dec 21;7:307. [Version 1] doi: 10.12688/wellcomeopenres.18660.1 (PMC10288160; doi:10.12688/wellcomeopenres.18660.1)
Supplement: Peer review history

# Peer review: Reviewer response for version 1

**DOI:** 10.21956/wellcomeopenres.20693.r59841

**Published:** 2023-06-22

**Authors:** Jin Sun

**Affiliations:** 1 Ocean University of China, Qingdao, Shandong, China

Annelida is a speciose phylum but with very few genomes reported. In Annelida, most of the genomes assembled are in the subclass Sedentaria, while there are even fewer genomes reported in Errantia. The genome of *Lepidonotus clava* is, therefore, an important contribution to this subclass. The overall genome assembly seems very high-quality. My only concern is regarding the Hi-C scaffolding. To me, the chromosome boundary is not very sharp, which may request further manual curation.

Are sufficient details of methods and materials provided to allow replication by others?

Yes

Is the rationale for creating the dataset(s) clearly described?

Yes

Are the datasets clearly presented in a useable and accessible format?

Yes

Are the protocols appropriate and is the work technically sound?

Partly

Reviewer Expertise:

Deep-sea biology, genomics, phylogenetics

I confirm that I have read this submission and believe that I have an appropriate level of expertise to confirm that it is of an acceptable scientific standard.

# Peer review: Reviewer response for version 1

**DOI:** 10.21956/wellcomeopenres.20693.r59836

**Published:** 2023-06-12

**Authors:** José María Martín-Durán

**Affiliations:** 1 Queen Mary University of London, London, England, UK

This is the genome note for the first chromosome-level genome assembly for Polynoidae (Annelida). *Lepidonotus clava* is a common segmented worm in the British islands and elsewhere. In Annelida, *Lepidonotus* is a member of the large Errantia clade, for which there is an urgent need for chromosome-level genome assemblies. Therefore, this dataset will help with phylum-wide comparative genomic analyses while providing a valuable resource to study a common member of the British marine macrofauna. The methods are state-of-the-art, and the resulting assembly is of the highest quality. I don't have any comments or concerns about this note.

Are sufficient details of methods and materials provided to allow replication by others?

Yes

Is the rationale for creating the dataset(s) clearly described?

Yes

Are the datasets clearly presented in a useable and accessible format?

Yes

Are the protocols appropriate and is the work technically sound?

Yes

Reviewer Expertise:

Evolutionary genomics, evolutionary comparative developmental biology, Annelid genomics

I confirm that I have read this submission and believe that I have an appropriate level of expertise to confirm that it is of an acceptable scientific standard.
